# Superoxide Dismutases in Eukaryotic Microorganisms: Four Case Studies

**DOI:** 10.3390/antiox11020188

**Published:** 2022-01-19

**Authors:** Alvaro de Obeso Fernandez del Valle, Christian Quintus Scheckhuber

**Affiliations:** Departamento de Bioingeniería, Escuela de Ingeniería y Ciencias, Tecnologico de Monterrey, Ave. Eugenio Garza Sada 2501, Monterrey 64849, Mexico; adeobeso@tec.mx

**Keywords:** *Acanthamoeba*, *Aspergillus*, development, pathogenicity, *Podospora* *anserina*, reactive oxygen species, superoxide dismutase, *Trypanosoma*

## Abstract

Various components in the cell are responsible for maintaining physiological levels of reactive oxygen species (ROS). Several different enzymes exist that can convert or degrade ROS; among them are the superoxide dismutases (SODs). If left unchecked, ROS can cause damage that leads to pathology, can contribute to aging, and may, ultimately, cause death. SODs are responsible for converting superoxide anions to hydrogen peroxide by dismutation. Here we review the role of different SODs on the development and pathogenicity of various eukaryotic microorganisms relevant to human health. These include the fungal aging model, *Podospora anserina*; various members of the genus *Aspergillus* that can potentially cause aspergillosis; the agents of diseases such as Chagas and sleeping disease, *Trypanosoma* *cruzi* and *Trypanosoma* *brucei*, respectively; and, finally, pathogenic amoebae, such as *Acanthamoeba* spp. In these organisms, SODs fulfill essential and often regulatory functions that come into play during processes such as the development, host infection, propagation, and control of gene expression. We explore the contribution of SODs and their related factors in these microorganisms, which have an established role in health and disease.

## 1. Introduction

Superoxide dismutases (SOD) are antioxidant metalloenzymes that dismutate O_2_^−^ into molecular oxygen and hydrogen peroxide (H_2_O_2_), thus, eliminating superoxide radicals. They are key players in defending cells from reactive oxygen species (ROS) during an infection of pathogens [1]. ROS, like superoxide, hydrogen peroxide, hydroxyl radicals, and singlet oxygen, among others, are usually toxic to the cell in elevated concentrations because of their high reactivity with biologically relevant molecules, e.g., proteins, nucleic acids, and lipids. However, in physiological concentrations, some of them are also important regulators of cellular signaling processes. Oxidative stress and its careful management are also important as it is one of the most common means of defense employed by the immune system when combating invading pathogens. As such, ROS are components of the direct and indirect antimicrobial immune response [2]. Furthermore, SODs can be regarded as a crucial defense against pathogens that survive attacks by the immune system of the host during an infection. Therefore, they tend to be the ideal drug target for different therapies [3]. In evolutionary terms, SODs show little structural homology, indicating the convergent evolution of this group of enzymes [4,5]. SODs even have different functions depending on their location within the cell and the organism.

**Figure 1:**
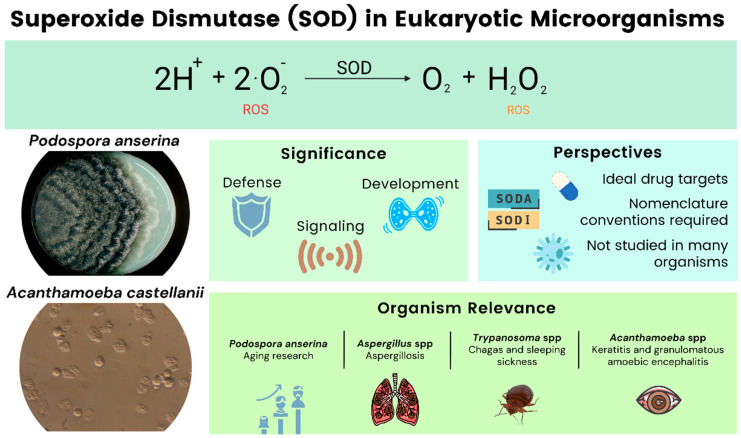
Overview of the article and the significance of SODs in eukaryotic microorganisms.

In this review, we provide an overview of SODs from four different microbial eukaryotes: *Podospora anserina*, *Aspergillus* spp., *Trypanosoma* spp., and *Acanthamoeba* spp. Our objective is to present several examples of microorganisms important to human health, in which research on SODs has unveiled important insights into their developmental processes or on other fundamental biological processes. The filamentous ascomycete *P. anserina* serves as a model for aging while the other organisms are mostly pathogenic. Various species of Aspergilli are grouped and discussed in one section. We also include data on the protists, *Trypanosoma* spp. and *Acanthamoeba* spp. We aim to provide a useful overview that aids the reader in understanding the state of knowledge and the opportunities for research regarding SODs and microbial diversity (Figure 1). At this point we would like to note that the SOD nomenclature is not standardized across the literature, and our work reflects this fact. In naming SODs, we generally use the nomenclature found in the references.

## 2. Fungi

In this section, the findings on oxidative stress in general, and SODs in particular, from a variety of fungi are reported. Although not a pathogen, research on *P. anserina* shows an impact on human health and disease by its use as a valuable model for studying the process of biological aging. A variety of *Aspergillus* species are being reviewed, as oxidative stress and antioxidant defense are the key regulators for the pathogenicity of most of these molds.

### 2.1. Podospora anserina: A Model System for Biological Aging

**Figure 2:**
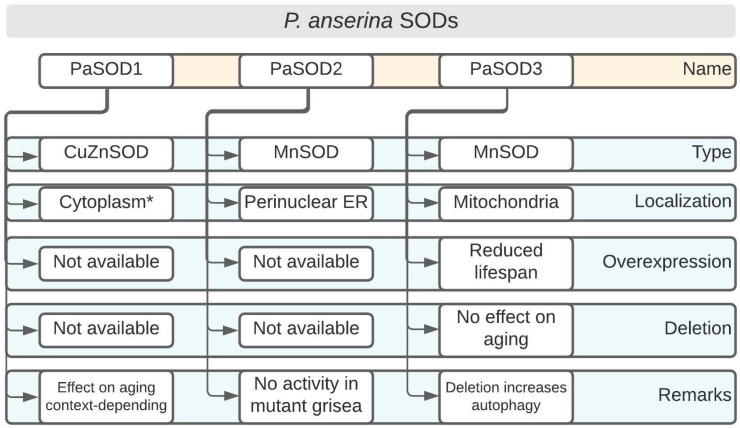
*Podospora anserina* SOD overview. * Further cellular localizations such as intermembrane space of mitochondria are likely. Overexpression and deletion denote genetic manipulations of the corresponding SOD-encoding genes. ER: endoplasmic reticulum.

Biological aging is defined as the continuous loss of vitality and viability, with a concomitant increase in morbidity and mortality [6]. Although there have been tremendous efforts to understand the aging process, there is still no consensus on how aging and its unwanted ‘side-effects’ can be reduced or countered. Rather, simple model systems have been utilized to foster our understanding of how processes, such as senescence and aging, mechanistically work. Fungi, for example, are attractive organisms on which to study the time-dependent changes that lead, ultimately, to organismal death [7,8]. In addition to the unicellular baker’s yeast (*Saccharomyces cerevisiae*), a considerable effort has been invested into characterizing aging at the molecular and cellular levels in the filamentous ascomycete, *P. anserina*, which is a close relative of the more well-known *Neurospora crassa* [9,10,11,12]. Among the studied pathways, the formation and detoxification of harmful ROS in *P. anserina* are particularly well-characterized [13,14]. According to the Free Radical Theory of Aging, ROS are causal agents of the aging process [15,16]. Although *P. anserina* is an obligate aerobe, mitochondrial defects are not necessarily lethal. In addition to the conventional complex IV (Cytochrome *c* oxidase, COX), the fungus can use an alternative oxidase (AOX) for maintaining its viability in case COX activity becomes compromised [17]. AOX-dependent respiration was found to lead to the decreased production of ROS in plants and fungi [18,19]. Supporting this line of evidence is the observation that numerous long-lived mutants of *P. anserina* utilize AOX, but not COX, as terminal oxidase [17,20]. In this section, we will focus on the role of SODs on *P. anserina* development (Figure 2).

#### 2.1.1. PaSOD1: A Differentially Regulated CuZnSOD in Certain Long-Lived Mutants

Two long-lived *P. anserina* mutants, that display interesting differences regarding their SOD activity profiles, will be briefly discussed here. The findings show that, depending on the genetic context, these enzymes might be potentially linked to a lifespan extension. SOD activity was assessed in the mutants, grisea and ex1, and was compared to the wild type (WT), which exhibits a normal lifespan [17,21]. In the WT, the activity of the mostly cytoplasmic CuZnSOD, PaSOD1, (Figure 2) increased strongly with age, whereas the activity of PaSOD2, a MnSOD which is either ER-associated or secreted [22], decreased [17]. The mutant ex1, in which AOX was induced due to a deletion in the mtDNA that led to the loss of the *CoI* gene (encoding the first subunit of COX), seemed to exclusively employ PaSOD1 [21]. In mutant grisea, a gene encoding a copper-modulated transcription factor (GRISEA) is functionally inactivated by mutation [23]. This mutant experienced severely depleted cellular copper levels [24]. Therefore, the activity of copper-containing proteins, such as COX and PaSOD1, is almost undetectable [21,25]. Importantly, putative target genes of the transcription factor GRISEA are, at most, very weakly expressed. Among these is *PaCtr3* (encoding a high-affinity copper transporter of the plasma membrane), which contributes to the severely reduced copper levels in grisea. In the WT, the differential activity of PaSOD1 pointed to a redistribution of cellular copper during aging where the amount of available copper in the cytoplasm increased. This is an example of a clear developmental regulation of PaSOD1 activity at the protein level.

In addition, PaSOD1 seems to play a crucial role in extension of the lifespan of the *PaCox17*::ble mutant [26]. In this mutant, the putative copper chaperone, PaCOX17, was deleted by gene replacement. The mutant displays an increased lifespan compared to the WT; even after 320 days, 40 out of the 60 cultures were still alive, whereas WT isolates have a lifespan of two to three weeks. PaSOD1 levels are highly upregulated in *PaCox17*::ble, suggesting an improved defense against superoxide anions [26].

Zintel et al. determined that the size of PaSOD1, through an in silico analysis, was 15.8 kDa [22]. Furthermore, PaSOD1 possesses two conserved CuZnSOD signatures. A predominantly cytoplasmic localization was revealed by the construction of a strain that synthesizes PaSOD1::GFP [22].

#### 2.1.2. PaSOD2: The Enigmatic MnSOD Is Inactivated in the Long-Lived Mutant grisea

Not all SODs are characterized equally in *P. anserina*. Here, PaSOD2 is introduced, which offers interesting perspectives for future research to elucidate its potential roles in the developmental processes of *P. anserina*, such as aging. 

PaSOD2 has an apparent molecular weight of 26.8 kDa and it contains putative manganese/iron binding motifs [22]. Originally, PaSOD2 was reported to represent a mitochondrial SOD [21,25], although it lacks a clear mitochondrial targeting sequence [22]. Later work showed that PaSOD2 is present in the perinuclear endoplasmic reticulum [22] (Figure 2). The role of a mitochondrial SOD is taken by PaSOD3 [22], which will be discussed below.

As mentioned previously, PaSOD2 activity decreases during aging in *P. anserina* WT strains [17]. *PaSod2* was originally thought to be a putative target gene of the copper-modulated transcription factor, GRISEA, because it is not detected in isolates of the mutant [17]. Due to the redistribution of cellular copper during the aging of *P. anserina,* GRISEA was supposed to be inactivated, leading to transcriptional inactivation of *PaSod2* [21]. Later research using transcriptional profiling showed that *PaSod2* was transcribed at WT levels in mutant grisea when copper was added to the growth medium; thus, it is not a target gene of GRISEA [27].

#### 2.1.3. PaSOD3: Increasing the Content of This Mitochondrial MnSOD, but Not Its Ablation, Has Effects on Aging 

This SOD is, by far, the best studied of the three identified family members of *P. anserina* (Figure 2). Mutants in which *PaSod3* levels were experimentally modulated revealed some unexpected findings, while cleary illustrating that PaSOD3 is linked to other important surveillance and quality control systems in *P. anserina*. PaSOD3 might also influence several processes by modulating ROS levels (superoxide anion and hydrogen peroxide) that are required for signaling, underscoring the role of ROS as cellular messengers.

PaSOD3 constitutes a mitochondrial SOD with a deduced molecular weight of 25.5 kDa. The enzyme contains a putative mitochondrial targeting sequence and was experimentally demonstrated to reside in mitochondria [22]. The impact of PaSOD3 on aging was studied by utilizing mutants in which *PaSod3* was either constitutively overexpressed or deleted [22]. ∆*PaSod3* strains were hypersensitive to compounds that generated superoxide anions, such as paraquat, which was expected. In contrast, the sensitivity to hydrogen peroxide was not altered in ∆*PaSod3*. However, the overexpression of *PaSod3* led to a significant growth reduction compared to the WT control, even without any additional stressors. Unexpectedly, these strains were more sensitive to both paraquat and hydrogen peroxide when these compounds were added to the growth medium [22]. Regarding the aging process, ∆*PaSod3* did not show any significant differences compared to the WT control. On the other hand, *PaSod3* overexpressors were short-lived when grown under standard conditions, reaching only around 75% of the median lifespan of the WT [22]. This can be explained, firstly, by the protein levels of the peroxiredoxin PaPRX1, a mitochondrial peroxidase that detoxifies hydrogen peroxide which was strongly reduced in *PaSod3* overexpressors, suggesting a compromised defense against ROS. Secondly, several proteins responsible for the quality control of mitochondria, such as matrix proteases PaCLPP and PaLON, were almost absent, or they exhibited altered protein patterns, due to their incomplete processing or degradation, respectively [22]. Thirdly, the mitochondrial heat shock protein PaHSP60 was clearly upregulated and proteolytically activated in the *PaSod3* overexpressing strains [22]. The authors suggest that the elevated PaSOD3 levels led to the generation of high doses of hydrogen peroxide in mitochondria, which was subsequently converted to the highly reactive hydroxyl radical. The latter is formed by Fenton chemistry involving hydrogen peroxide and various metal ions and it has a highly destructive potential due to its extremely high reactivity with lipids, nucleic acids, and proteins [28].

To better understand the phenotypic effects of the increased *PaSod3* expression, a modeling strategy was employed that supported the hypothesis that excess hydrogen peroxide generated by PaSOD3 was responsible for the damaging effects [29]. The computational studies suggested that the levels of the PaSOD3 cofactor, Mn^2+^, were elevated by a factor of 80 in the *PaSod3* overexpressors. This result led to a study of the role of manganese supplementation of the growth medium of the WT and the *PaSod3* overexpressors on phenotypic parameters [30]. Interestingly, the supplementation of the growth medium with MnSO_4_, even in small amounts (20 µM), led to a reversion of the *PaSod3*_OEx mutant phenotype. In addition to showing WT-like growth rates, the *PaSod3* overexpressors had restored the formation of aerial hyphae and fertility. Regarding aging, MnSO_4_ concentrations of 80 µM allowed the *PaSod3*_OEx strains to reach median lifespans indistinguishable from those of the WTs [30]. 

Importantly, the quantification of the total SOD activity (PaSOD1, PaSOD2 and PaSOD3) demonstrated no significant differences in whole cell extracts or isolated mitochondria, regardless of whether Mn was added to the growth medium or not. The authors concluded that a general limitation of manganese on SOD activity in *PaSod3* overexpressors did not occur [30].

However, in contrast to the WT, the Mn-supplemented *PaSod3* overexpressors demonstrated elevated levels of peroxidase and catalase activities in addition to the upregulated protein levels of the peroxiredoxin, PaPRX. Further results suggested that a hitherto unknown Mn-dependent protein contributed to an improved degradation rate of hydrogen peroxide [30].

PaSOD3 protein levels were shown to be controlled by the protein, PaRCF1 [31]. PaRCF1 belongs to the HIG1 (‘hypoxia-inducible gene 1’) family of proteins, which play an important role in the assembly and organization of the mitochondrial respiratory chain [32]. A deleted mutant of *PaRcf1*, ∆*PaRcf1*, contains only a fraction of PaSOD3 proteins (approximately 20%) compared to the WT, but its activity appears not to be affected [31]. However, ∆*PaRcf1* is hypersensitive to the addition of the redox cycler, paraquat, into the medium in comparison to the WT. Additionally, this mutant fails to maintain a normal growth rate, is sterile, and is marked by a decreased median lifespan. It is important to note that in ∆*PaRcf1*, PaSOD3, as well as other factors of maintenance and cellular quality control decreased, e.g., PaLON, PaPRX and PaCLP [31]. Thus, the altered phenotype of ∆*PaRcf1* is likely the result of several deficiencies. 

As mentioned above, the signaling function of ROS is altered in *PaSod3* deletion strains [30]. It is known that ROS are modulators of various cellular processes; among these is the controlled cellular ’self-eating’, or autophagy [33,34]. Usually, ROS are activators of autophagy, which acts as a system to assist in maintaining cellular homeostasis [35]. It was shown that the ’unexpected healthy phenotype’ of ∆*PaSod3* was due to the induction of autophagy (Figure 2) [36]. The authors demonstrated this by analyzing the number of autophagosomes using the marker *Gfp*-*PaAtg8*. These increased, even in juvenile stages. Increased autophagy (especially mitophagy) is important for ∆*PaSod3* survival because the ∆*PaSod3* ∆*Paatg1* double knockout strain reveals severe phenotypic deficiencies, such as reductions in both the growth rate and lifespan [36].

SODs are enzymes that degrade superoxide anions. However, the generation of superoxide anions is an important process for growth and development in fungi. *P. anserina* and many other organisms express genes that encode plasma membrane NADPH oxidases, which are sources of superoxide production [37,38]. 

It was found that genes encoding NADPH oxidases (*Nox*) are exclusively present in the genomes of multicellular organisms, regardless of their phylogenetic origin, pointing to a role of controlled superoxide production in differentiation processes [39]. Deactivating the *P. anserina* gene, *PaNox1*, leads to several defects, such as the reduced pigmentation of the mycelium, the insufficient formation of aerial hyphae and, most importantly, the compromised formation of perithecia (fruiting bodies). PaNOX1 functions in cellular signaling upstream of the mitogen-activated protein kinase kinase kinase (MAPKKK), PaASK1 [40]. Meanwhile, the deletion of *PaNox2*, a second NADPH oxidase isoform in *P. anserina*, demonstrates that this gene is essential for the germination of ascospores [40]. In summary, the regulated secretion of superoxide anions and peroxide during the life cycle is controlled by proteins, including PaNOX1, PaNOX2 and PaASK1 [40].

Enzymes such as PaSOD2 are potentially secreted, degrading superoxide anions, thereby modulating the O_2_^−^-mediated developmental signaling, although, for now, this is speculative and requires further investigation.

The work on the role of SODs in the developmental process of *P. anserina* firmly positions these proteins as crucial components of an elaborate network, controlling several vital processes, such as growth, fertility, the intracellular communication of biological quality control pathways and, ultimately, aging.

### 2.2. Aspergillus spp.: The Often Pathogenic Fungi Affecting Human Health

The genus *Aspergillus* contains several hundred members. Most of them have a cosmopolitan distribution. Many species of this genus are known for their pathogenicity, e.g., *A. fumigatus* and *A. flavus*. Others display only mild pathogenicity and are of industrial importance, such as *A. niger*, *A. oryzae*, and *A. terreus*. *A. nidulans* is a valuable model organism for studying eukaryotic cell biology. 

#### 2.2.1. *A. fumigatus*: One of the Most Important Fungal Pathogens

*A. fumigatus* is one of the best characterized fungal pathogens. In the environment, it plays a crucial role in the recycling of carbon and nitrogen. Due to its abundant sporulation, it releases a huge quantity of conidia into the air that is potentially problematic for people with a compromised immune system. Aspergillosis symptoms first begin in the lungs. In severely predisposed individuals, any organ can be targeted [41]. *A. fumigatus* is “the most important opportunistic human pathogen among phylogenetically closely related aspergilli” [42]. SOD research using *A. fumigatus* has revealed several important aspects on how these enzymes co-modulate pathogenicity. Several key findings are outlined below.

The *A. fumigatus* genome encodes four putative SODs: AfSOD1p, a cytoplasmic CuZnSOD; AfSOD2p, a mitochondrial MnSOD; AfSOD3p, a cytoplasmic MnSOD; and AfSOD4p, which displays a MnSOD domain at its C-terminus [43]. 

The genes encoding AfSOD1p and AfSOD2p are strongly expressed in *A. fumigatus* conidia. In contrast, AfSOD3p is found mostly in the mycelium. Although the gene encoding AfSOD4p is only weakly expressed, its deletion is lethal to the fungus. However, it remains to be experimentally shown as to whether AfSOD4p is a *bona fide* SOD or not [43]. Research showed that the decreased resistance to temperature stress and the ROS-generating menadione are the hallmarks of *AfSOD1* and *AfSOD2* deletion. The construction of a triple deletion mutant, ∆*AfSOD1-3*, resulted in a complex phenotype. The deletion strains were very sensitive to menadione and were killed by the macrophages in the alveoli of immunocompetent mice. Unexpectedly, virulence, per se, was not distinguishable from the control parental strain in immunocompromised mice [43]. Copper insertion is a critical step for CuZnSOD maturation. In baker’s yeast, this step is mediated by the copper chaperone, Ccs1p [44]. Recently, the role of the Ccs1p ortholog from *A. fumigatus* has been studied in detail [45]. The resulting mutant of a deletion of *ccsA* was characterized by the elevated accumulation of ROS and a higher degree of sensitivity to oxidative stress. Importantly, the conserved CXC motif was essential for the interaction between CcsA, SODA, and the adaptation to oxidative stress. SODC, a MnSOD, complemented the defects exhibited by *ccsA* or *sodA* deletions, if overexpressed, or when Mn^2+^ was added to the growth medium [45]. Interestingly, the abrogation of the CcsA–SODA complex did not lead to an altered virulence of the fungus.

In addition to building blocks of the cell wall, such as galactomannan, chitin synthetases, and immune response modulators (*rodA/hyp1* and *pksP/alb1*), several proteins mediating oxidative stress defense have been implicated in pathogenicity, e.g., catalases (Cat1p and Cat2p) and SODs (MnSOD and CuZnSOD) [46].

In *A. fumigatus*, the expression of genes encoding SODs were found to be controlled by the regulator of the G-protein signaling protein, RgsC, which is highly conserved in ascomycetes [47]. Furthermore, the deletion of the *rgsC* gene led to a pleiotropic phenotype. The described hallmarks include compromised growth, asexual development, the reduced ROS tolerance of conidia, and the decreased virulence in the wax moth [47]. The phenotype of the mutant could be related to the compromised ROS signaling because the gene expressions and enzymatic activities of catalases and SODs were severely decreased [47].

Under the conditions of iron-deprivation, *A. fumigatus* was found to transcriptionally upregulate genes encoding CuZnSOD and other iron-independent antioxidant proteins [42,48]. This is not unexpected, because several important antioxidant enzymes depend on the levels of available iron as a cofactor, such as catalases and heme peroxidases [49]. Conditions of low iron and oxidative stress are encountered by *A. fumigatus* when it colonizes the human body [42]. It was found, through a transcriptome analysis, that iron deprivation increases oxidative stress susceptibility and pharmacologically targeting these iron-independent antioxidant defense systems might offer the potential to effectively combat fungal infections. Purified CuZnSOD from *A. fumigatus* has been successfully employed in the detection of aspergillosis because it has a high level of immune reactivity to the sera of patients [50].

#### 2.2.2. *A. flavus*: Spoiling Food by Toxin Production

*A. flavus* is a human pathogen that can cause aspergillosis. Additionally, it can spoil several food sources such as cereal grains, legumes, and tree nuts. The consumption of contaminated feed can increase the chance for liver cancer because *A. flavus* produces the mycotoxin, Aflatoxin B1 [51]. Aflatoxin B1 is one of the most dangerous mycotoxins. Several results point towards a connection between aflatoxin synthesis and the production and detoxification of ROS. Aflatoxin B1 toxicity can be ameliorated by piperine, which is usually found in certain peppers [52,53]. It was shown that piperine not only downregulated the set of genes that led to aflatoxin toxicity, but it also positively influenced the concentrations of antioxidants in *A. flavus* [54]. Several genes that encode antioxidant proteins, including members of the SOD family, were significantly upregulated upon piperine treatment. There is a clear correlation between levels of ROS and the capability of *A. flavus* to synthesize aflatoxin [55,56,57]. However, a study showed that the superoxide generator, menadione, was capable of decreasing aflatoxin production in *A. flavus* NRRL3357 [58]. In this study, SOD activity decreased in the presence of menadione. A study aimed at deciphering the interplay between oxidative stress, superoxide activity, and aflatoxin biosynthesis found that the redox cycler and the superoxide generator, paraquat, at low concentrations, inhibited aflatoxin production [59]. The transcription of the regulator gene *aflR* and the aflatoxin biosynthetic cluster genes were downregulated by the paraquat treatment. Although the addition of purified CuZnSOD to the culture medium counteracted the paraquat-mediated increase in superoxide production by *A. flavus*, aflatoxin biosynthesis was not restored, because the CuZnSOD protein itself displayed an inhibitory effect [59]. By contrast, cytosolic and mitochondrial superoxide production was capable of downregulating aflatoxin production by decreasing *aflR* expression. The mechanism by which supplementing CuZnSOD to the growth medium resulted in a reduction of aflatoxin formation is not yet clear, but it likely does not involve the detoxification of superoxide anions [59].

*A. flavus*, being an opportunistic pathogen, was found to be susceptible to the puroindoline B (PINB) protein [60]. PINB is capable of severely compromising the fungus, evidenced by withering of the mycelium, the damage to cell membranes, and the disruption of the tricarboxylic acid cycle. It is noteworthy that in addition to catalase, SOD activity was found to be reduced. As a result, the GSH/GSSG ratio was reduced and the levels of ROS were elevated [60]. These results demonstrate that antioxidant enzymes are among the best targets for controlling pathogens such as *A. flavus*.

A variety of antifungal peptides for improving food safety have been evaluated [61]. The expression of MnSOD was found to be affected at the transcript level, as evidenced by RT-qPCR. Intracellular levels of ROS decreased in the fungi subjected to the peptides. ABTS and DPPH assays were used to demonstrate that the peptides harbor antioxidant activities. The expression of the genes in the aflatoxin biosynthesis cluster was downregulated, as well as the conidiation of the mycelia [61]. In summary, the analyzed antifungal peptides are promising inhibitors of toxicity from Aspergilli, such as *A. flavus*.

MnSOD was found to be important for *A. flavus* when it exploits maize as a source of nutrients [62]. Four trophic conditions were analyzed in this work: (a) Czapek Dox (CD) medium in flasks, (b) Czapek Dox (CD) medium in flasks containing injured maize kernels within a closed dialysis tube, (c) autoclaved maize kernels, and (d) maize ears in the field. Under all tested conditions, the mutant without MnSOD was shown to grow slower than the WT control. Under growth condition (d), aflatoxin production was significantly decreased [62]. The authors hypothesize that the ROS produced at the point of contact between ∆*sod* and its host severely affected the fitness of this mutant. Furthermore, the increased ROS production in the mitochondria of the mutant might cause molecular damage to essential components of the TCA cycle, such as aconitase. This might force the mutant to reroute its resources to maintain growth and antioxidant defense so that it can no longer adequately support the biosynthesis of aflatoxin [62].

#### 2.2.3. *A. parasiticus*: An Important Aflatoxin Producer

*A. parasiticus*, like *A. flavus*, is known as a potent producer of aflatoxin [63]. The relationship between aflatoxin biosynthesis and cellular ROS levels was investigated in detail in the *A. parasiticus* strain, SU-1, and its aflatoxin-negative mutant, AFS10, in detail [64]. SU-1 handled intracellular ROS much more effectively than AFS10, which is likely due to the efficient transcriptional activation of five genes encoding members of the SOD family. However, supplementing the growth medium of AFS10 with aflatoxin led to a noticeable reduction of ROS without changing the expression of the genes encoding SODs. In conclusion, aflatoxin biosynthesis aids *A. parasiticus* in coping with oxidative stress. This occurs dependent of the *aflR* regulator gene and the mycotoxin itself [64]. It would be interesting to analyze whether *A. flavus* demonstrates a similar behavior.

#### 2.2.4. *A. terreus*: The Emerging Pathogen of Human Health

In contrast to several other members of the genus *Aspergillus*, such as the aforementioned *A. fumigatus*, the mold *A. terreus* is not as common an agent of aspergillosis [65]. However, in recent years, the fungus is increasingly receiving attention because the cases in which it causes disease are steadily increasing [65]. *A. terreus* shows high resistance to the antibiotic, amphotericin B, which is problematic for the treatment of infections. However, there are also *A. terreus* isolates that do display a susceptibility to amphotericin B. Jukic et al. compared pathogenicity between an amphotericin B-resistant and a susceptible *A. terreus* strain [66]. Resistant strains responded much more effectively to amphotericin B treatment than sensitive strains, shown by their strongly induced transcript levels of *Sod2* and the gene encoding catalase. Importantly, inhibiting these antioxidant proteins confers a susceptibility to amphotericin B [66]. Therefore, enzymes of the oxidative stress response should be regarded as interesting targets for treating infections by *A. terreus* and, possibly, by other Aspergilli as well.

#### 2.2.5. *A. nidulans*: A Valuable Model System for the Study of Eukaryotic Cell Biology

*Aspergillus* spp. are capable of producing enormous quantities of conidia. This ability is linked to several factors involving mitochondrial function. ROS and the modulation of their cellular levels by antioxidant enzymes are integrated into the network that controls the production of conidia. Due to the ease of constructing genetically altered strains, *A. nidulans* is highly valuable for the study of developmental processes, such as conidiation. 

Here, we give an example in which the potential targets for counteracting asexual reproduction, by focusing on the characterization of mitochondrial quality control systems, were identified [67]. The components were *aodA*, an alternative oxidase in the mitochondrial respiratory chain; *dnmA*, a dynamin-like protein involved in organelle fission; *pimA*, a mitochondrial LON protease; and the mitochondrial SOD, MnSOD. The mutant phenotypes of *A. nidulans* with deletions and overexpressions of these genes were compared to experimental data from *P. anserina* research. Importantly, many differences in phenotypic aspects were detected between *A. nidulans* and *P. anserina* [67]. For example, several of the phenotypes were only observed sporadically, or were distributed without an emerging pattern among the gene overexpression and deletion mutants. The only common denominator was a clearly impaired amount and viability of conidiospores. Importantly, the decreased production and viability of conidiospores was observed in all gene deletion mutants, pointing out the clear role of mitochondrial functionality in asexual reproduction. One of the main mitochondrial differences is that in *P. anserina*, mtDNA is subject to gross reorganizations during aging [10]. Several of the aforementioned gene orthologs might, thus, affect mitochondrial biology differently in *P. anserina* than in *A. nidulans*. Lastly, it should be kept in mind that both species are not extremely closely related, with *P. anserina* belonging to the taxonomic unit of Sordariomycetes and *A. nidulans* to Eurotiomycetes, which might also help to explain at least some of the observed differences.

#### 2.2.6. *A. glaucus*: An Interesting Platform for the Production of Pharmaceuticals

There is a constant demand to isolate improved and more stable enzymes that have superior properties, with SODs being no exception. One example is the cold-adapated *A. glaucus* that enabled the isolation of an SOD with such properties. Among the Aspergilli, *A. glaucus* is better adapted to more extreme environmental conditions than most of its relatives [68]. For example, *A. glaucus* strain 363 was reported to contain a cold-adapted CuZnSOD that is highly active at low temperatures, such as those lower than 10 °C [69]. This antioxidant could be very useful in the protection of sperm cells in the field of assisted reproductive technology [69]. In addition, *A. glaucus* produces mycotoxins, such as aspergiolide A, that are being evaluated as potential anti-cancer agents [70].

## 3. Protists

In this section we focus on two different protists. Protists are a very diverse polyphyletic group, and the diversity of SODs demonstrates this fact. For example, in the slime mold *Dictyostelium discoideum*, SODs serve as starvation signals that are causative of the multicellular stage, and gene overexpression can inhibit multicellularity [71]. In the case of the malaria-causing unicellular protozoan parasite *Plasmodium falciparum*, Fe-SOD has been suggested as a highly selective target for antiparasitic drugs [72]. Furthermore, SODs and pyruvate have been linked to metronidazole resistance in *Entamoeba histolytica*, meaning that there could be a link to drug resistance, as it is associated with other forms of stress [73,74]. Several protozoans have Fe-SOD, which are regularly found in prokaryotes, but are also present in archaeal organisms and plants. In evolutionary terms, Fe-SODs are the most ancient SODs [4]. It is important to note that not all protists present SODs, as in the case of the giardiasis-causing flagellate parasite *Giardia duodenalis* [75,76].

Two different genera were chosen to exemplify the role of SODs in potentially pathogenic protists: *Trypanosoma* and *Acanthamoeba*. 

### 3.1. Trypanosoma spp.: Pathogenic Organisms Responsible for Typanosomiasis

Trypanosomes are hemoflagellate aerobic protozoans which cause African and American trypanosomiasis, also known as sleeping sickness and Chagas disease, respectively [77]. Trypanosomiasis is listed by the World Health Organization (WHO) as one of seventeen neglected tropical diseases [78]. Trypanosomes have a complex life cycle that includes a trypomastigote that lives in insects (depending on the species) and is capable of infecting mammals. Inside mammals, and, therefore, humans, the trypomastigote invades cells and becomes an amastigote that starts dividing. Finally, amastigotes become bloodstream trypomastigotes that lyse the cells and infect new cells. When the infected person comes into contact with another insect, the parasite becomes an epimastigote that survives in the insect’s gut [77]. Among the *Trypanosoma* species, the most relevant are *T. cruzi* (Chagas disease) and *T. brucei* (sleeping sickness). However, *T. evansi* is the causative agent of “surra”, an important disease that affects camels, horses, and dogs [79]. Additionally, *T. vivax* is the most common trypanosome in Africa, and causes animal trypanosomiasis, or nagana disease, in cattle [80,81]. As *Trypanosoma* can become intracellular parasites, SODs play an important role in their survival as a defense against oxidative stress and are, therefore, ideal therapy targets. Notably, some of the most common enzymes against oxidative stress are catalases and glutathione peroxidase. These are missing from trypanosomatids, making the role of SOD even more important [82,83]. Next, we examine the role of SODs in *T. cruzi* and *T. brucei*. 

#### 3.1.1. *Trypanosoma cruzi*: A Causative Agent of Chagas Disease

Chagas disease, or American trypanosomiasis, is caused by *T. cruzi* and occurs mainly in Latin America and parts of the United States. *T. cruzi* is transmitted by triatomine bugs that are also known as kissing bugs. The disease can be asymptomatic, acute, or chronic, and can lead to death [77].

Macrophages produce superoxide toxicity as a first line of defense against *T. cruzi* [84]. There are several defenses against oxidative stress in *T. cruzi*. Among them are: arginase, isocitrate dehydrogenase, the glutathione peroxidase-like protein, trypanothione reductase, Fe-SOD, the acetylornithine deacetylase-like protein, Cytochrome *b*_5_ reductase, and thiol-dependent reductase [85]. Several SODs have been identified in *T. cruzi*. The first was an iron-dependent SOD [86]. Soon after, two SODs were cloned and characterized: Fe-SODA and Fe-SODB [87]. Fe-SODA is constitutively expressed, while Fe-SODB is overexpressed during the epimastigote stage [87]. Later, four different SOD activities were characterized in epimastigotes (SOD I, II, III and IV). SODI and SODIII appear to be present as dimers, as the resulting sizes do not match any sequences in the trypanosomatid genome and are mainly cytosolic [88]. Two of these Fe-SOD-encoding genes are found as multiple repeating units across the genome of *T. cruzi* [87].

*T. cruzi* produces cytosolic SOD to defend itself from superoxides. An Fe-SOD enriched *T. cruzi* showed an almost two-fold rate of survival compared to the WT when challenged with macrophages [84]. Fe-SODB can detoxify cytosolic superoxide derived from macrophages, preventing damage and increasing the virulence of *T. cruzi* [89]. 

Additionally, SODs have an important role in the *Trypanosoma* life cycle. SODA plays a role in amastigogenesis, as shown by a 70% increase in the expression of the gene after 60 min of trypomastigote induction [90]. The increased expression of *SODA* decreased the number of apoptotic amastigotes. This means it plays a role in survival from antioxidant-based immune responses [91]. 

Being intracellular pathogens, trypanosomes are highly sensitive to ROS. Therefore, SODs are interesting targets for drug development [92]. *T cruzi* hosts, transfected with a Fe-SOD (overexpressed 5- to 8-fold), demonstrated increased sensitivity against trypanocidal agents such as benznidazole and gentian violet, probably due to imbalances in the antioxidant defenses of the parasite [86]. *T. cruzi* strains that are resistant to benznidazole presented an increased expression of the gene encoding SODA [93]. Benznidazole-resistant strains decreased SODB synthesis and increased that of SODA [94]. SOD inhibition in *T. cruzi*, by the derivatives of aza-scorpiand-like macrocycles, showed antichagasic properties [95]. Additionally, 1,4-bis(alkylamino)benzo[*g*]phthalazines 1–4 containing one or two imidazole rings showed an inhibition of the parasite’s Fe-SOD and a significant decrease in amastigote and trypomastigote numbers without affecting human Cu-Fe-SOD [96]. Deoxymikanolide is a sesquiterpene lactone that showed decreased SOD activity of up to 40% after 24 h. However, according to the authors, the antiparasitic activity of the deoxymikanolide was more strongly related to a decrease in thiol groups that the sesquiterpene causes. The SOD activity was, therefore, probably related to the damage caused by the intracellular oxidative state [97]. Further examples are the two compounds for the leishmanicidal [1,2,3]Triazolo[1,5-a]pyridinium salt compound that showed trypanocidal effects through the inhibition of Fe-SOD activity [98]. Drug susceptibility may, therefore, be strain-dependent [94].

SODs have been suggested as diagnostic tools against Chagas disease. A study that identified *T. cruzi* antibodies, using Fe-SOD as the antigen, showed a 99.13% sensitivity and a 96.01% specificity for the diagnosis of Chagas disease in 969 Mexican blood donors [99]. Additionally, 1029 individuals were tested in Queretaro, Mexico, through Western blot, ELISA, and indirect haemagglutination for *T. cruzi* SODs. A seroprevalence value of 8.16% was obtained, which is higher than those previously reported in Chagas disease endemic regions [100]. Similar experiments were performed to identify *T. cruzi* antibodies in cats (7.36% seroprevalence) and dogs (with a seroprevalence ranging from 10.74%, 17.1%, and 21.34%, depending on the region and the study) across the Yucatan Peninsula [101,102,103]. Lastly, a similar study was performed that testd dogs in Tabasco and they presented a 3.36% serological prevalence in domiciled dogs out of 119 sera [104].

#### 3.1.2. *Trypanosoma brucei*: Causative Agent of Sleeping Sickness

Sleeping sickness, or African trypanosomiasis, is caused by *T. brucei* gambiense or *T. brucei* rhodesiense. These parasites cause chronic disease and are transmitted by tsetse flies. Sleeping sickness is fatal without treatment [77].

Four SOD have been identified in *T. brucei*, three of which are Fe-SODs [105]. The SODs are TbSODA, TbSODB1, TbSODB2, and TbSODC, where Tb refers to the organism. TbSODB1 is mainly cytosolic, while TbSODB2 is mainly glycosomal. TbSODA and TbSODC are mitochondrial [106]. A phylogenetic analysis of 106 SODs indicated that trypanosomatid SODs have been acquired through more than one horizontal transfer event [106].

TbSODB1 plays an important role in the defense against superoxide-inducing drugs, as it has been shown that the deletion of the gene increased its sensitivity to nifurtimox and benznidazole [107] The downregulation of glycosomal TbSODB2 corresponded to an increase in cell death among the population [105]. The aqueous extract of fruit pulp of the African tree *Adansonia digitata* showed effective trypanocidal effects through the inhibition of SODs against *T. brucei* in albino rats [108]. Other extracts or compounds studied as potential therapies, using SOD as a target, include: zinc and selenium [109], ɑ-tocopherol [110], and tetradentated pyridine-based manganese complexes such as Cpd2 and Cpd3 [111].

As is the case in *T. cruzi*, SODs play a role in the life cycle of *T. brucei*. A type of Fe-SOD was cloned and was identified as playing a crucial role in *T. brucei*, which is only required in the proliferation stages of the organism to eliminate superoxide radicals produced during this developmental stage [112].

### 3.2. Acanthamoeba spp.: The Pathogenic Free Living Amoebae

*Acanthamoeba* are free-living amoeboid organisms with a worldwide distribution. They are capable of producing diseases such as granulomatous amoebic encephalitis and *Acanthamoeba* keratitis [113]. The main species of *Acanthamoeba* is *A. castellanii*. As a free-living organism with pathogenic capabilities, *Acanthamoeba* must be capable of surviving with different oxidative conditions. SODs in *Acanthamoeba* have not been extensively researched. However, there are several reviews related to the biology and pathogenicity of *Acanthamoeba* that have highlighted their importance [114,115,116].

Two SODs have been identified in *Acanthamoeba* so far; Fe-SOD (approximately 50 kDa) and CuZn-SOD (approximately 38 kDa). These *Acanthamoeba* SODs are found in the cytoplasm and in the detergent-extractable phase [114,117]. The Fe-SOD is formed by a 2.33 Å single crystal with well-conserved fold and active-site residues [118]. A third mitochondrial Mn-SOD has been related to mitochondrial bioenergetics [119].

During phagocytosis, *Acanthamoeba* produces superoxides, similar to phagocytes. due to a “respiratory burst” of oxidase that aids in the phagocytosis of bacteria [120,121]. *A. castellanii* has superoxide-generating NADPH oxidase that kills and lyses bacteria, similar to *D. discoideum* [122]. This process is comparable to what macrophages employ to lyse and kill bacteria [116]. *Acanthamoeba* utilizes SODs as an environmental adaptation with antioxidant properties [123]. *Acanthamoeba* also requires SODs to be able to adapt to oxidative conditions in hosts and to defend itself from oxidative death by immune effector cells [124]. As they act as antioxidants and anti-inflammatory agents, they have been identified as potential virulence factors [117]. SOD activity was shown to be lower in the virulent species of the genus *Acanthamoeba*, ranging from 40–65 units/mg of protein in non-pathogenic strains, to 21–23 units/mg of protein in pathogenic strains [125]. Fe-SOD is vital for *Acanthamoeba* survival, as it helps in the defense against endogenous oxidative stress and oxidative death by immune effector cells. SODs could be targets for chemotherapy and immunodiagnoses [117].

*Acanthamoeba* shows the plasticity of mitochondrial bioenergetics during batch cultures where the in vitro production of ROS decreases. This high plasticity ensures the survival of high quality cysts under stress-decreasing ROS without altering SOD production [119]. The 26 kDa Mn-SOD activity stayed close to 100% after 96 h in a batch culture to protect against ROS production [119].

## 4. Perspectives/Conclusions

The study of SODs plays an important role in comprehending the biology of organisms such as *P. anserina*, *Aspergillus*, *Trypanosoma*, and *Acanthamoeba*. SODs have been extensively studied in some clades but have been slightly neglected in some regions of the evolutionary tree. We aimed to highlight the potential of these enzymes for studying, understanding, and possibly even controlling these types of organisms. Even though we limit our review to four genera, SODs play an important role in a great variety of microorganisms. Table 1 proposes some literature for the reader who wants to enhance their understanding on the role of SODs in other relevant eukaryotic microorganisms which have not been addressed in the present review.

In general, ROS were viewed for a long time as largely unavoidable toxic by-products of several metabolic pathways. However, as already mentioned in the introduction, it has become clear that they also serve essential functions in regulating metabolism as signaling molecules. Several of the early theories on the causes of organismic aging pointed to ROS as agents of molecular damage, cumulating eventually in pathogenicity and death [126]. However, more recent work, especially from the nematode *Caenorhabditis elegans*, as well as in rodents, paints a different picture [127]. ROS can be regarded as mediators and coordinators of the stress response systems that become triggered during aging. Among these are the fusion and fission of organelles, such as mitochondria and peroxisomes, the proteolytic degradation of damaged enzymes, and the coordinated removal of compromised organelles (autophagy) [128,129,130]. Antioxidant enzymes, similar to the SODs in which this contribution is focused, allow the conversion of highly reactive superoxide radicals to become less reactive and more long-lived, for example hydrogen peroxide, which can pass greater distances within the cell [131]. In general, due to their high reactivity, most ROS exist only for a very short time in the crowded environment of the cell, approximately 100 ns for singlet oxygen, for example [132]. However, similar to hydrogen peroxide, superoxide anions can interact specifically with target enzymes. This has been demonstrated for the soluble form of guanylate cyclase, with which the cGMP-producing activity is inhibited by O_2_^−^ [133]. As such, SODs not only serve a protective role during cellular life and development, but they are also integrated into elaborate signaling networks. Unraveling these networks is challenging but necessary to understand various biological processes outlined in our review, such as growth, sexual and asexual propagation, metabolite biosynthesis, pathogenicity, and, ultimately, aging.

Additionally, we would like to stress the importance of a standardized nomenclature for naming and distinguishing SODs. Currently, a plethora of naming schemes are being used by the scientific community. Like valuable efforts towards unifying the nomenclature of cell death factors [141], meta- and paracaspases [142], and autophagy-related genes [143], this step is necessary, in our opinion, to avoid confusion when it comes to naming SODs.

Finally, the study of SODs and their potential roles as regulators of developmental processes is limited to a certain set of organisms. Certainly, it could be very valuable to initiate research projects to unravel the importance of SOD activity in organisms that have not received much attention, in this regard, so far.

## Figures and Tables

**Table 1:** Selected literature for relevant eukaryotic microorganisms not covered in this review.

Organism	Significance	Reference
Protists		
*Dictyostelium discoideum*	Social slime mold, studies of multicellularity	[71]
*Entamoeba histolytica*	Pathogen of amebiasis	[76]
*Giardia lamblia*	Lacks SODs, agent of intestine infection	[75]
*Plasmodium falciparum*	Pathogen of malaria	[134]
*Toxoplasma gondii*	Pathogen of toxoplasmosis	[135]
Fungi		
*Neurospora crassa*	Circadian rhythms, epigenetics, gene silencing, cell polarity	[136]
*Penicillium chrysogenum/rubrum*	Antibiotics and enzyme production	[137]
*Pichia pastoris*	Heterologous protein production	[138]
*Saccharomyces cerevisiae*	Many aspects of basic and applied science	[44]
*Schizosaccharomyces pombe*	DNA damage, repair, and replication	[139]
*Sordaria macrospora*	Fruiting body development	[140]
